# Remote estimation of rice LAI based on Fourier spectrum texture from UAV image

**DOI:** 10.1186/s13007-019-0507-8

**Published:** 2019-11-01

**Authors:** Bo Duan, Yating Liu, Yan Gong, Yi Peng, Xianting Wu, Renshan Zhu, Shenghui Fang

**Affiliations:** 1 School of Remote Sensing and Information Engineering, Wuhan University, Wuhan, China; 2 College of Life Sciences, Wuhan University, Wuhan, China; 3 Lab for Remote Sensing of Crop Phenotyping, Wuhan University, Wuhan, China

**Keywords:** Remote sensing, UAV, Rice LAI, Vegetation index, Fourier spectrum texture

## Abstract

**Background:**

The accurate estimation of rice LAI is particularly important to monitor rice growth status. Remote sensing, as a non-destructive measurement technology, has been proved to be useful for estimating vegetation growth parameters, especially at large scale. With the development of unmanned aerial vehicles (UAVs), this novel remote sensing platform has been widely used to provide remote sensing images which have much higher spatial resolution. Previous reports have shown that the spectral feature of remote sensing images could be an effective indicator to estimate vegetation growth parameters. However, the texture feature of high-resolution remote sensing images is rarely employed for this purpose. Besides, the physical mechanism between the texture feature and vegetation growth parameters is still unclear.

**Results:**

In this study, a Fourier spectrum texture based on the UAV Image was developed to estimate rice LAI. And the relationship between Fourier spectrum texture and rice LAI was also analyzed. The results showed that Fourier spectrum texture could improve the accuracy of rice LAI estimation.

**Conclusions:**

In conclusion, the texture feature of high-resolution remote sensing images may be more effective in rice LAI estimation than the spectral feature.

## Background

Leaf area index (LAI) is a key canopy structure parameter that related to the photosynthesis, respiration, and transpiration of vegetation [1]. The accurate estimation of crop LAI is of significance to monitor the health and nutrient status, thus providing effective technical support in fertilizer application and water management for precision agriculture [2].

Remote sensing (RS) is a technology which can obtain the information about an object without making physical contact with the object [3]. Therefore, RS has a distinct advantage in monitoring vegetation growth due to its non-destructive characteristic. It has been proved that RS could efficiently acquire canopy spectral data which contains a large of information on the canopy interaction with solar radiation such as vegetation absorption and scattering [4]. Vegetation reflectance and vegetation index (VI) have been developed as main spectral features to evaluate vegetation growth. The vegetation reflectance is closely related to vegetation growth. In the visible range, a relatively lower reflectance value appeared due to the strong light absorption of leaf pigments [5]. In the near-infrared range (NIR), vegetation reflectance becomes obviously higher affected by thick plant tissues and canopy structure [6]. And VI is mathematical combinations of different spectral ranges mostly in the visible and NIR regions [7]. The main purpose of VI is to enhance the vegetation information contained in spectral reflectance data. In previous study, various VIs have been proposed to retrieve biophysical parameters such as LAI [8, 9], chlorophyll content [10–12] and biomass [13, 14]. Generally, regression algorithms are used to develop the relationship between VI and biophysical parameters. Although the VI-based regression algorithms are simple and easily available, most VIs are not robust when applied across different regions. In this way, a more sophisticated statistical technique—the machine learning method, has been considered [15]. For example, Bacour et al. [16] established a LAI estimation model with MERIS satellite reflectance at 11 bands by neural network. Verrelst et al. [17] applied Gaussian process machine learning method to retrieve chlorophyll content with 62 bands CHRIS reflectance. Machine learning methods can make full use of reflectance information at different bands and obtain approximate complex non-linear functions, and they are more robust and adaptive than VI-based regression algorithms [18].

In essence, the spectral feature contains a limited amount of information acquired by RS images. Besides the spectral feature, RS images provide more abundant texture information related to vegetation growth. The texture feature should also be considered when the RS image is used to retrieve vegetation growth parameters. Some research has been conducted to analyze the relationship between image texture features and vegetation growth. Nichol et al. [19] found that the texture parameter of high-resolution optical sensors can improve forest biomass estimation. And the similar result was also found by Sarker [20]. Zhang et al. [21] combined the object-based texture features with a neural network to examine the contribution of the spatial information for vegetation mapping and found that the texture features could improve the accuracy of vegetation mapping. Correlations between the texture feature and vegetation growth were revealed in many studies, but the texture feature was always used to establish the statistical algorithm or used as the inputs for machine learning methods. The physical mechanism of relating texture feature to vegetation growth is still unclear. In addition, there is little research comparing the use of the spectral feature and the texture feature for estimating vegetation growth parameters.

Recently, unmanned aerial vehicles (UAVs) are increasingly used as an innovative RS platform for agricultural applications [22–24]. In comparison to traditional satellite platform, the flexibility of changing UAV flight altitude and attitude can give us an easy access to data with different spatial resolutions as required by users. This is particularly beneficial for precision agriculture by providing the image with selected resolution for detailed observations on different crop growth. For example, Jin et al. [25] proposed a method to estimate wheat density using images taken by UAV at very low altitude (3–7 m); López-Granados et al. [26] determined weed distributions in croplands using UAV images collected at different flying heights. With high-spatial-resolution UAV data, more detailed texture information about vegetation growth can be derived from images. Therefore, when UAV RS technology is used to monitor vegetation growth, it is extremely important to take account of the texture feature of UAV images.

This study explores to improve rice LAI estimation using Fourier spectrum texture from UAV images. The first objective is to compare using canopy reflectance and VI for rice LAI estimation. The second objective is to analyze the physical mechanism of relating Fourier spectrum texture to LAI. The final objective is to compare the performances of using the spectral feature and the texture feature to estimate rice LAI.

## Materials and methods

### Study area

**Fig. 1:**
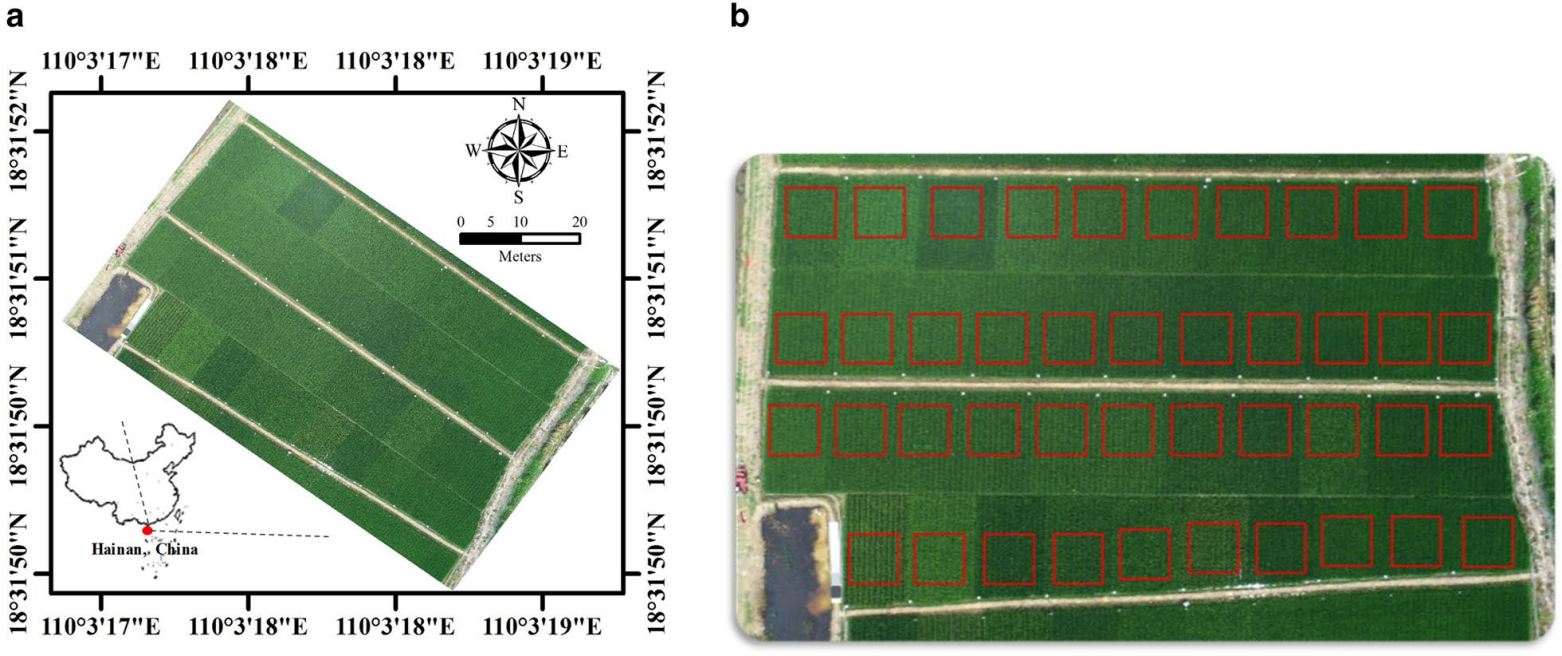
**a** The study area and **b** the region of interest (ROI) in 42 rice plots

**Fig. 2:**
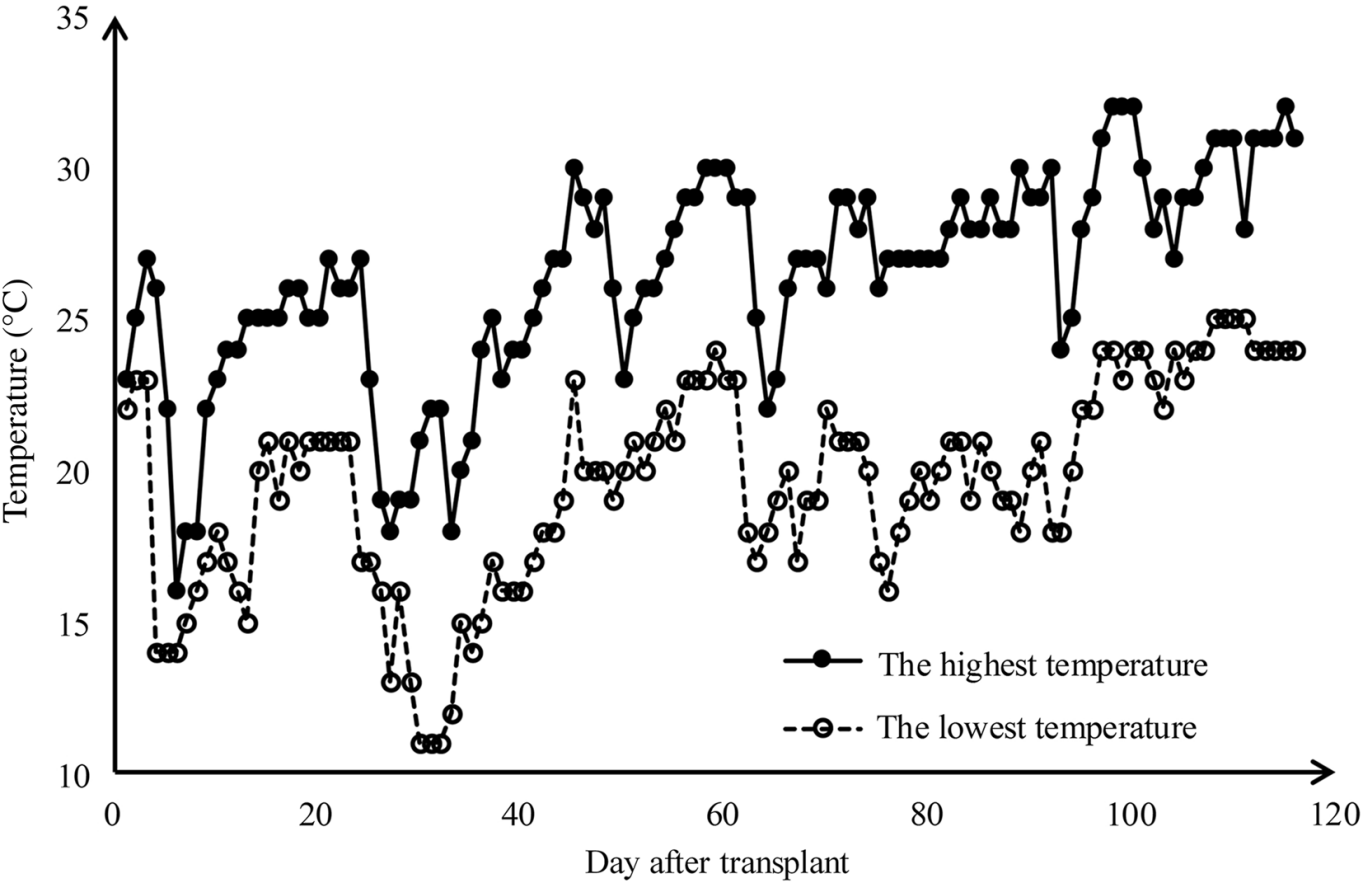
The air temperature change after the transplant of rice plant

The study site was located at the Hybrid Rice Experiment and Research Base of Wuhan University near Lingshui city, HaiNan Province, China (18°31′47.1″N 110°03′34.9″E). The terrain of study area is flat, and there is a tropical marine climate with high temperature throughout the year. There were 42 representative hybrid rice cultivars planted in different field plots. The plots were of the same size about 70 m^2^ but were different in shape—Fig. 1a. The plant density and nitrogen application for these plots were same. In order to distinguish these plots in the UAV image, several white boards were erected at the edge of plots. The experiment was conducted for a single season from December 2017 to May 2018. All rice cultivars were sown on December 10th 2017 and transplanted on January 5th 2018 with the transplanting density of 15,000 plants/ha. The air temperature change during the whole growth period of rice was shown at Fig. 2 and there was not a large amount of precipitation in this period. Six field experiments were conducted on February 4th, February 25th, March 9th, March 19th, March 31th and April 17th respectively. In each field experiment, one UAV flight was arranged to obtain the image of all rice plots. After the UAV flight, the corresponding ground LAI measurements were carried out immediately.

### In-situ LAI data collection

In this study, a destructive sampling measurement was used to collect rice LAI data in different plots. For each plot, three bundles were randomly dug out from soil with root, placed in a bucket full of water and taken to the laboratory. LAI measurements were taken after returning the samples to the laboratory immediately. The green leaves were separated from stems and panicle components. If a larger proportion of a leaf was yellow, it was recognized as a yellow leaf. The total green leaf area of each bundle was measured by LI-3000C leaf area meter (LI-COR, Lincoln, NE, United States) with the unit of square meter. And the average leaf area of three bundles represented the single plant leaf area in each plot. According to the planting density of rice, the LAI value of each plot was

1 $${\text{LAI}} = {\text{LA}}_{s} \times {\text{d}}$$

where LA_s_ was the single plant leaf area in each plot and ${\text{d}}$ was plant density in one square meter. For each of 42 plots, six LAI values were collected on different days and thus the sample size of LAI in this paper was 252.

### Canopy reflectance and vegetation index derived from the UAV image

In this study, an UAV equipped with a Mini-MCA system was used to acquire the image of study plots. The Mini-MCA system consisted of an array of twelve individual miniature digital cameras (Mini-MCA 12, Tetracam, Inc., Chatsworth, CA, United States). Each camera imager was equipped with a customer-specified band pass filter centered at the wavelength of 490 nm, 520 nm, 550 nm, 570 nm, 670 nm, 680 nm, 700 nm, 720 nm, 800 nm, 850 nm, 900 nm and 950 nm respectively. These spectral bands basically covered the visible to NIR region which were commonly employed to analyze vegetation growth-related parameters [27, 28]. The Mini-MCA system was fixed in the UAV by a gimbal which can help to compensate for the UAV movement (pitch and roll) during the flight and guarantee close to nadir image collection [29]. The UAV flight was conducted under clear skies with little cloud cover between 10 am and 2 pm local time when the changes in the solar zenith angle were minimal. The altitudes for UAV images acquisition was 200 m and the spatial resolution was 108.33 mm.

Since the MCA system had a significant camera mis-registration effect, the band-to-band registration was conducted in the laboratory prior to the flight so that corresponding pixels of each lens were spatially overlapping in the same focal plane. And an empirical linear correction method was applied to transform image digital number (DN) into surface reflectance (R) [30, 31]. Six calibration ground targets which had the constant reflectance were placed in the cameras’ field of view as the reference for image radiometric correction. These six calibration targets had the relatively constant reflectance of 0.03, 0.12, 0.24, 0.36, 0.56 and 0.80 respectively throughout the visible to NIR wavelengths. Based on the linear relationship between DN and R, the reflectance value was obtained

2 $$R_{\lambda } = DN_{\lambda } \times Gain_{\lambda } + Offset_{\lambda }$$

where $\lambda$ was the band wavelength of MCA camera; $R_{\lambda }$ and $DN_{\lambda }$ were the surface reflectance and digital number of a pixel at wavelength ${\lambda }$ respectively; $Gain_{\lambda }$ and $Offset_{\lambda }$ were gains and bias. For each wavelength ${\lambda }$, $Gain_{\lambda }$ and $Offset_{\lambda }$ can be calculated using the least-square method by R and DN values (referring to $DN_{0.03}$, $DN_{0.12}$, $DN_{0.24}$, $DN_{0.36}$, $DN_{0.56}$, and $DN_{0.80}$) of six calibration targets.

3 $$\left( {\begin{array}{*{20}c} {0.03} \\ {0.12} \\ {0.24} \\ {0.36} \\ {0.56} \\ {0.80} \\ \end{array} } \right) = \left( {\begin{array}{*{20}c} {DN_{0.03} } \\ {DN_{0.12} } \\ {DN_{0.24} } \\ {DN_{0.36} } \\ {DN_{0.56} } \\ {DN_{0.80} } \\ \end{array} } \right) \times Gain_{\lambda } + Offset_{\lambda }$$

After radiometric correction, the raw UAV images with DN values were transformed into reflectance images and then band math was employed to obtain VI images. Seven VIs were calculated in this study—Table 1.

**Table 1:** The Vegetation Indices tested in this study

Vegetation indices	Formula	References
Red-edge Chlorophyll Index (CI_rededge_)	R_800nm_/R_720nm_−1	Gitelson et al. [32]
Green-edge Chlorophyll Index (CI_green_)	R_800nm_/R_550nm_−1	Gitelson et al. [32]
Normalized Difference Vegetation Index (NDVI)	(R_800nm_−R_670nm_)/(R_800nm_ + R_670nm_)	Rouse et al. [33]
Normalized Difference Red edge (NDRE)	(R_800nm_−R_720nm_)/(R_800nm_ + R_720nm_)	Glenn et al. [34]
Visible Atmospherically Resistant Index (VARI)	(R_550nm_-R_670nm_)/(R_550nm_ + R_670nm_)	Gitelson et al. [35]
MERIS Terrestrial Chlorophyll Index (MTCI)	(R_800nm_−R_720nm_)/(R_720nm_−R_670nm_)	Dash and Curran [36]
Two-band Enhanced Vegetation Index (EVI2)	2.5(R_800nm_-R_670nm_)/(R_800nm_ + 2.4R_670nm_ + 1)	Jiang et al. [37]

We defined a rectangle in the image as the region of interest (ROI) of each rice plot—Fig. 1b. The rectangle was the standard square with a size of 41 × 41 pixels, and these squares were applied in reflectance image and VI image respectively. The average of all the per-pixel values within ROI was obtained as the plot level canopy reflectance and VI.

### Fourier spectrum texture extraction

Fourier transform is a common method of transforming images from the spatial domain into the frequency domain [38]. And in the frequency domain, the Fourier frequency spectrum is always employed to reflect the intensity of different frequency components. Generally, the characteristics of Fourier frequency spectrum contain a large of information on image texture. In this paper, the rectangle ring Fourier spectral energy percentage (FSEP) was used to represent the texture feature of rice field [39].

**Fig. 3:**
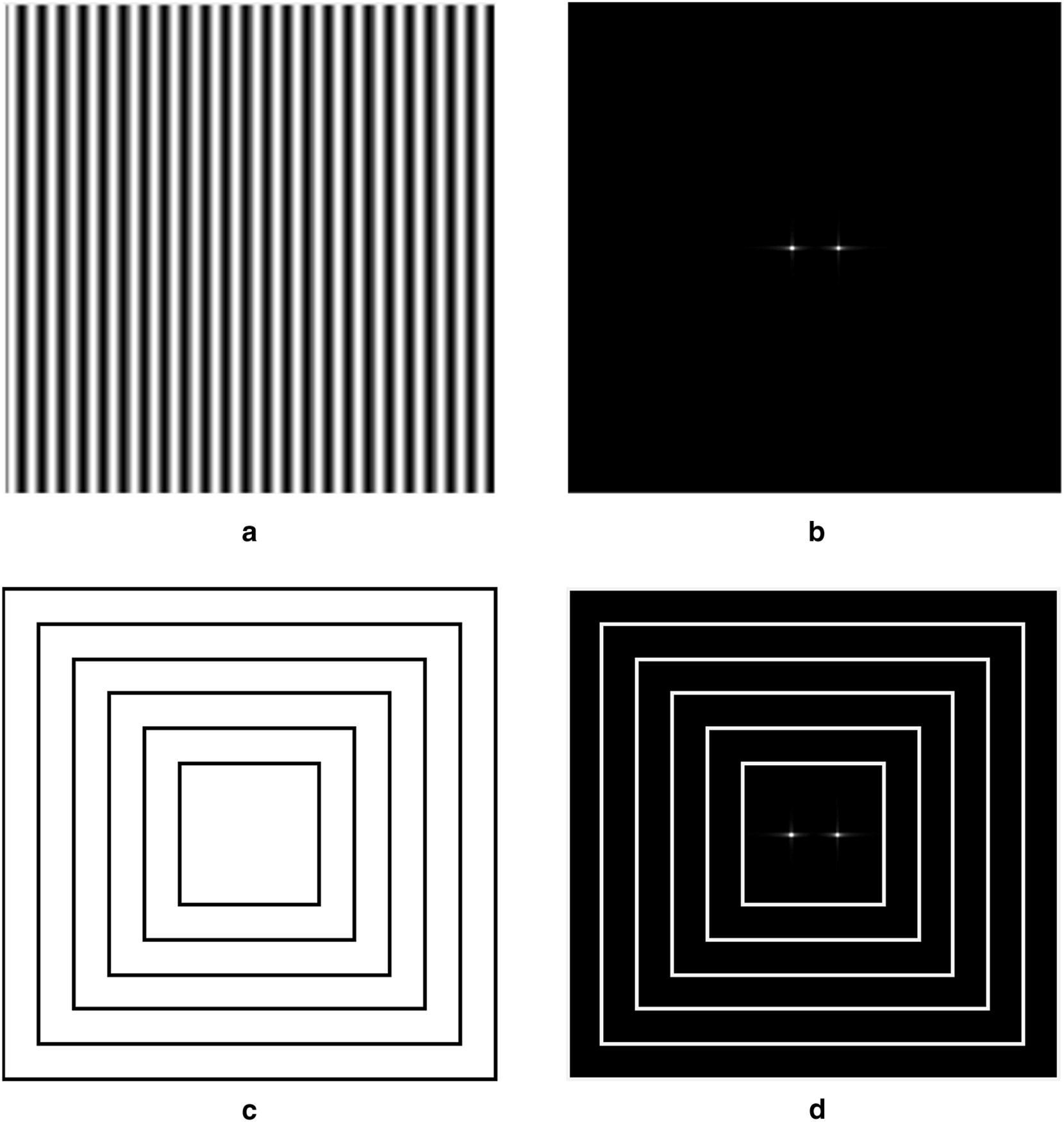
**a** The origin image of Fourier transform, **b** the Fourier energy spectrum, **c** the rectangle ring used in energy spectrum and **d** the Fourier spectral energy percentage of center ring

First of all, image was transformed into the frequency domain by Fourier transform. Since the image was the two dimensional discrete data, the transformation can be denoted as

4 $$F\left( {u,v} \right) = \mathop \sum \limits_{x = 1}^{M} \mathop \sum \limits_{y = 1}^{N} f\left( {x,y} \right)e^{{ - j2\pi \left( {\frac{ux}{M} + \frac{vy}{N}} \right)}} u = 1,2,\ldots,M;v = 1,2,\ldots,N$$

where f(x,y) was the digital image, x and y were the lateral axis and vertical axis of mage, M and N were the counts of row and column. F(u,v) was the Fourier frequency spectrum, and it was a complex function of two real frequency variables u and v. In the Fourier frequency spectrum image, u corresponded to lateral axis and v corresponded to vertical axis. Note that, the value of F(u,v) was a complex number and the energy spectrum of Fourier transform was

5 $$E\left( {u,v} \right) = R^{2} \left( {u,v} \right) + I^{2} \left( {u,v} \right)$$

where R(u,v) and I(u,v) were the real and imaginary parts of F(u,v) respectively. The energy spectrum was symmetrical about the center point of image due to the conjugate symmetry of Fourier transform and the energy spectrum image was the same size as the origin image—Fig. 3a, b. In this study, some rectangle rings in different sizes but in same shape were used to separate the Fourier energy spectrum—Fig. 3c and the percentage of center ring energy above the total energy of all rings was calculated to represent the texture feature. The rectangle ring started at the edge of the image and the distance between rectangle ring and image edge increased one pixel each time until the image cannot be separated—Fig. 3d. The FSEP was then defined as

6 $$FSEP = \frac{{E_{cen} }}{{\mathop \sum \nolimits_{u = 0}^{M} \mathop \sum \nolimits_{v = 0}^{N} E\left( {u,v} \right)}}$$

where E_cen_ was the energy of center ring and it was the sum of E(u,v) contained in center ring.

For each rice plot, the data contained in the rice plot ROI was extracted and saved as a two dimensional matrix. And then, the matrix was used to do Fourier transform and calculate the plot level FSEP.

### Data analysis and accuracy assessment

In this paper, we applied correlation analysis and regression analysis to describe and analyze the relationship between LAI and different remote sensing features extracted from the UAV image. Firstly, plot level canopy reflectance and VI were correlated with rice LAI directly. The Pearson correlation coefficient (r) was exhibited as the result of correlation analysis. And for regression analysis, the exponential regression model was used and the coefficient of determination (R^2^) was compared. Secondly, the same correlation analysis and regression analysis were also applied to analyze the relationship between LAI and different FSEPs extracted from different reflectance images and VI images. And we discussed the difference between spectral features and texture features. Finally, we established the rice LAI estimation model using Support Vector Machine Regression (SVR) [40] with the kernel function was radial basis function [41]. Forty-two rice plots were divided into two groups, one for SVR training (n = 30) and the other one for testing (n = 12). All the LAI samples of training plots were used to train SVR model. In this way, the LAI samples of the whole rice growth period attended to model establishment. Thus, the LAI training set included 180 samples and the LAI testing set included 72 samples. The algorithm was realized in MATLAB R2016a (MATLAB 2016a, s, Inc., Natick, MA, United States) with the LIBSVM package (https://www.csie.ntu.edu.tw/~cjlin/libsvm/). Some spectral parameters and FSEP parameters were tested as the input of SVR. To assess the estimation model, RMSE and R^2^ of estimated LAI and measured LAI in the LAI testing set were obtained.

## Results

### Correlations of LAI with canopy reflectance and vegetation index

**Fig. 4:**
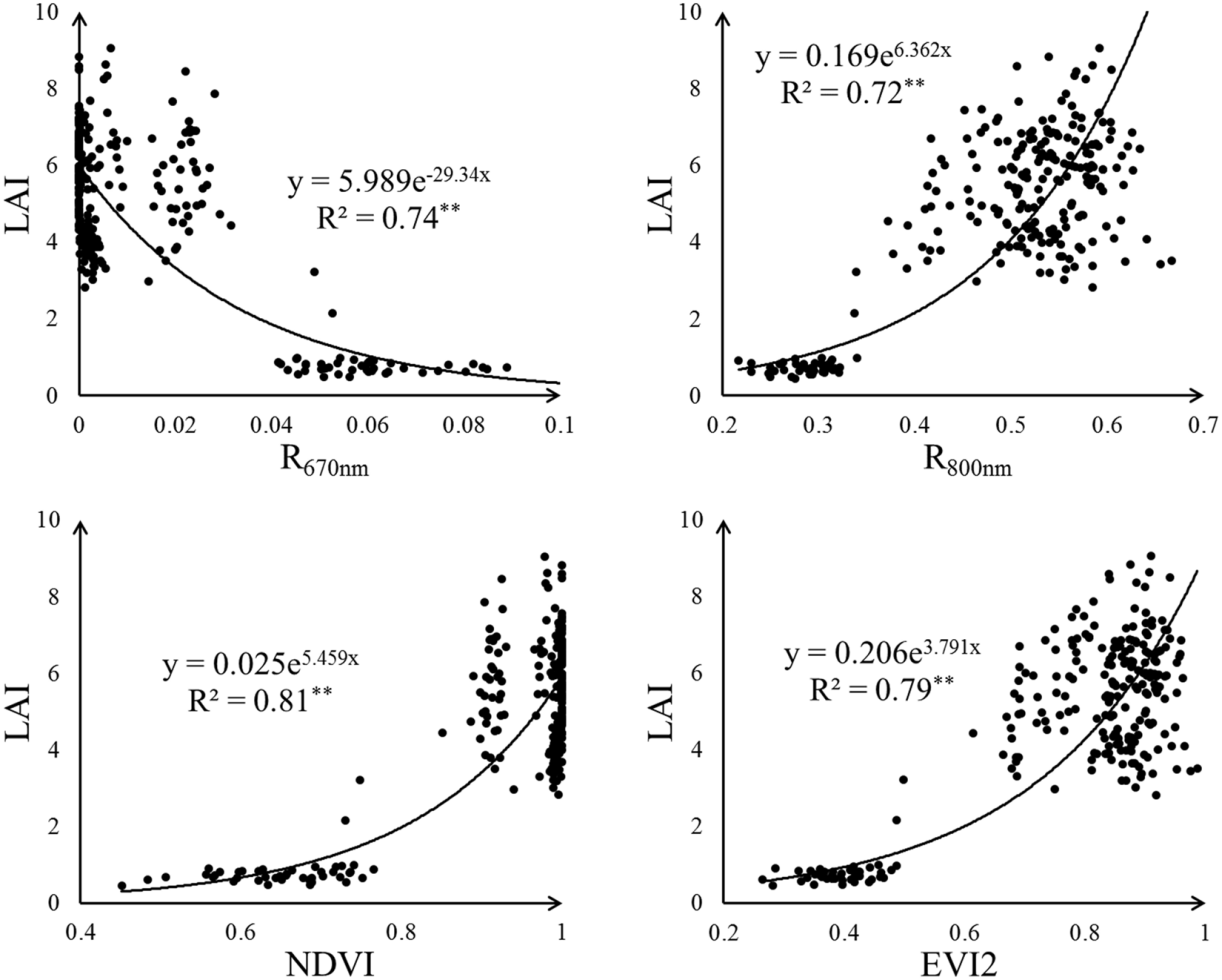
The result of regression analysis of LAI with R_670nm_, R_800nm_, NDVI and EVI2

To determine the relationship between rice LAI and spectral features derived from the UAV data, we conducted correlation analysis of LAI with canopy reflectance in different bands (reflectance in 550 nm, 670 nm, 720 nm and 800 nm, abbreviated as R_550nm_, R_670nm_, R_720nm_ and R_800nm_ respectively) and seven VIs. The results indicated that all the tested VIs were correlated positively with LAI but most reflectance bands exhibited a negative correlation with LAI except R_800nm_—Table 2. Among four reflectance bands, R_670nm_ and R_800nm_ showed the strong correlations with LAI (r was above 0.75), and R_720nm_ appeared a weakly correlation with LAI having the r value below 0.4. Generally, VIs exhibited stronger correlations with LAI and the r values of most VIs were above 0.65. Among all tested VIs, EVI2 and NDVI were the most relevant to LAI with the r value reaching 0.79. However, the regression analysis showed that NDVI had a significant saturation phenomenon and the value of R_670nm_ concentrated near 0—Fig. 4. Due to the saturation phenomenon of NDVI and the value aggregation of R_670nm_, they were not suitable for rice LAI estimation. Therefore, EVI2 and R_800nm_ were selected as the best spectral features to estimate rice LAI.

**Table 2:** The Pearson correlation coefficients (r) of LAI with reflectance and VI

	R_550nm_	R_670nm_	R_720nm_	R_800nm_	CI_rededge_	CI_green_	NDVI	NDRE	VARI	MTCI	EVI2
r	− 0.64^**^	− 0.75^**^	− 0.38^**^	0.77^**^	0.67^**^	0.52^**^	0.79^**^	0.74^**^	0.68^**^	0.67^**^	0.79^**^

** Correlation is significant at the 0.01 level (two-tailed)

### Fourier spectrum texture of rice field

**Fig. 5:**
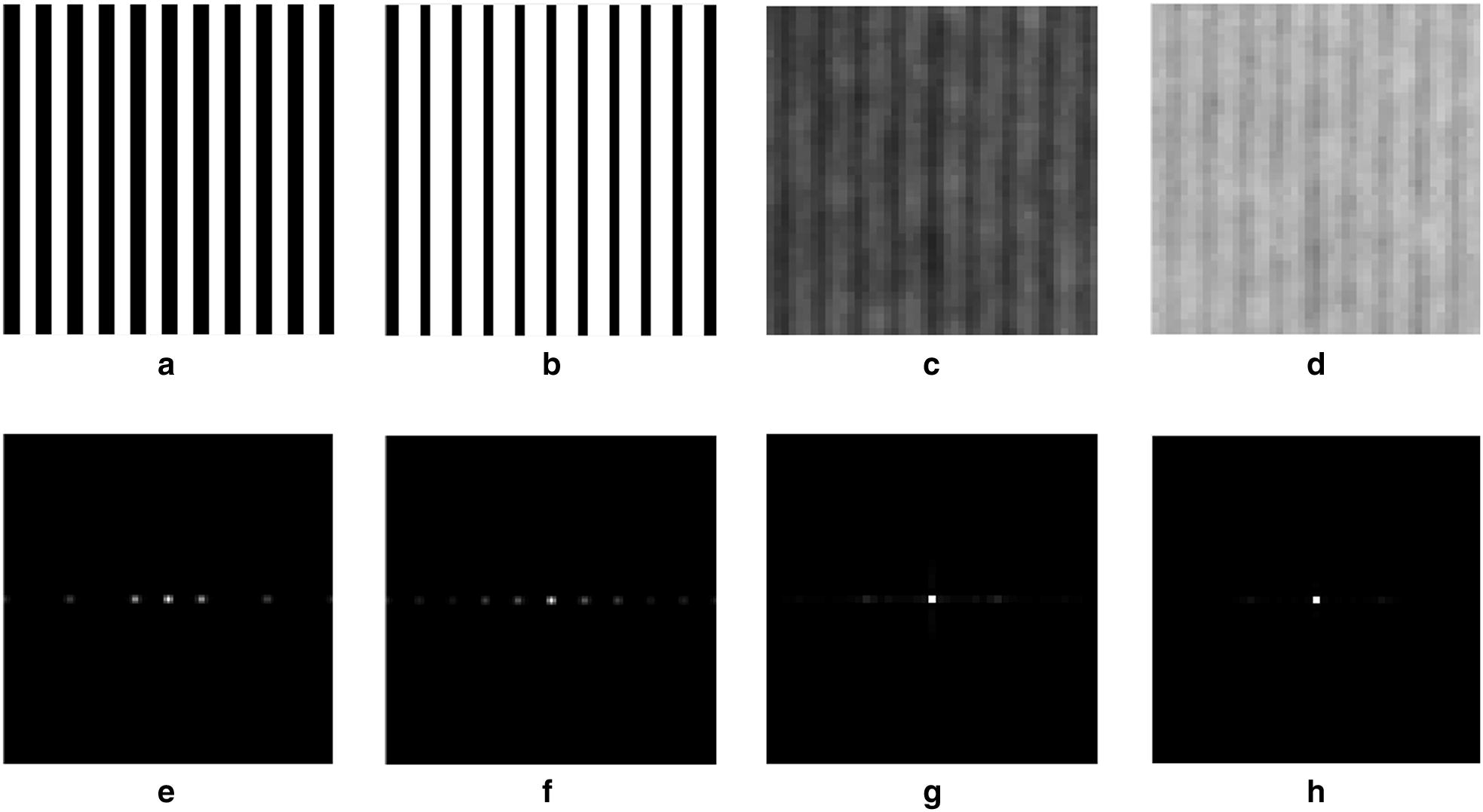
The origin images and their corresponding energy spectrum. The simulated images with **a** narrow stripe and **b** wide stripe, the actual UAV image of rice plot taken on **c** February 4th and **d** March 9th. **e**–**h** are the corresponding energy spectrum images of **a**, **b**, **c**, **d** respectively

In order to investigate the texture feature of rice field, Fourier transform was applied to transform the digital image from the spatial domain into the frequency domain and the Fourier energy spectrum was obtained to represent the texture feature of images. Firstly, two simulated images with white strips were used to analyze the difference of the Fourier energy spectrum between different images—Fig. 5a, b. The results showed that the high energy values of the Fourier energy spectrum were distributed in the center horizontal line. Similarly, the high energy value of the energy spectrum with wider stripes was closer to the midpoint of center line—Fig. 5e, f It can be inferred that the distribution of the energy spectrum was related to the stripe width. The wider the stripe, the more concentrated on center of the high energy value was. For further study, real UAV images of rice field were utilized. We selected two EVI2 images which focused on the same rice plot but were obtained on two different days—Fig. 5c, d. The image of Fig. 5c was got on February 4th and Fig. 5d was on March 9th. Generally, the LAI of rice on March 9th was higher than that on February 4th. The energy spectrum of UAV images was similar to that of simulated images and the high energy value also distributed in the center horizontal line—Fig. 5g, h Obviously, in Fig. 5g there were two darker bright spots on two sides of the brightest spot, but there was only one bright spot in Fig. 5h. It means that the high energy value of the energy spectrum was more concentrated on the center with the increase of rice LAI. Therefore, the characteristics of the Fourier energy spectrum were related to rice LAI, especially for the center region of energy spectrum.

### Relationship between LAI and Fourier spectrum texture

**Fig. 6:**
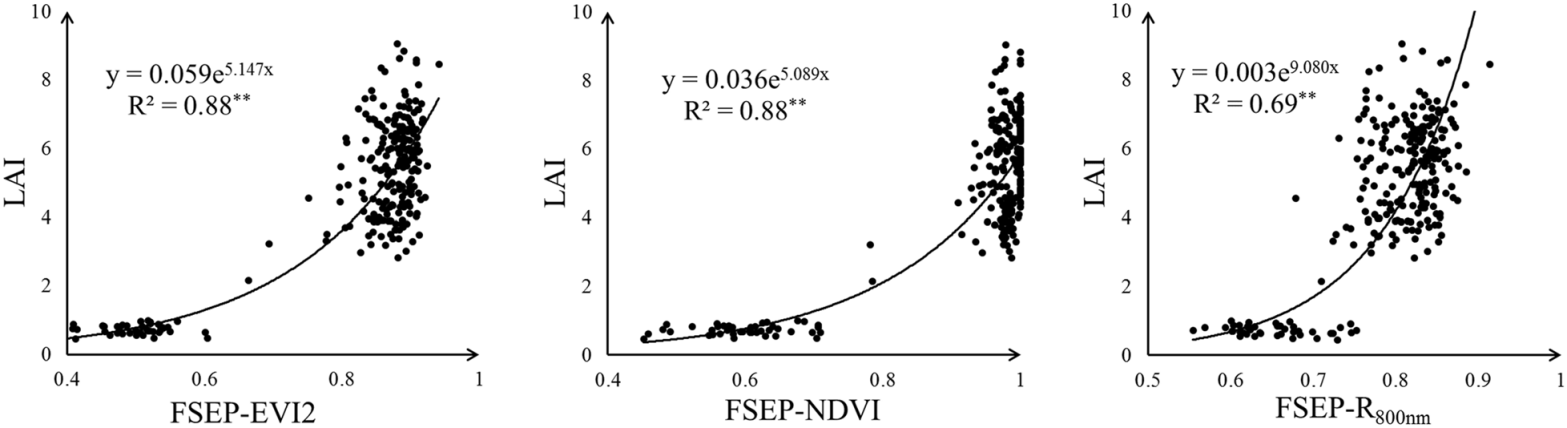
The result of regression analysis of LAI with FSEP-EVI2, FSEP-NDVI and FSEP-R_800nm_

To investigate the relationship between Fourier spectrum texture and rice LAI, we calculated a series of FSEP based on different reflectance and VI images. Firstly, LAI was directly correlated with these FSEPs and the Pearson correlation coefficients (r) were compared—Table 3. Generally, all VI-derived FSEPs produced higher r values with LAI than VIs. Especially for the VIs which had relatively lower r values, their FSEPs performed an obviously stronger correlation with LAI (such as CI_rededge_ and CI_green_). The results of correlation analysis indicated that VI-derived FSEPs correlated positively with LAI. Among all VI-derived FSEPs, FSEP-EVI2 still had the highest r value with LAI (r was 0.83). On the contrary, most reflectance-derived FSEPs exhibited a relatively weak correlation with LAI (r below 0.4) except FSEP-R_800nm_. Although FSEP-R_800nm_ showed a much stronger correlation with LAI (r was 0.75) than other reflectance-derived FSEPs, the r value of FSEP-R_800nm_ was a bit lower than that of R_800nm_ (r was 0.77—Table 2). The regression analysis showed that a significant saturation phenomenon still existed in FSEP-NDVI—Fig. 6. And FSEP-EVI2 had an extremely better goodness of fit with LAI (R^2^ was 0.88) than FSEP-R_800nm_ (R^2^ was 0.69). Therefore, the VI-derived FSEP was more suited to rice LAI estimation than the reflectance-derived FSEP.

**Table 3:** The Pearson correlation coefficients of LAI with FSEP based on reflectance and VI images

	FSEP-R_550nm_	FSEP-R_670nm_	FSEP-R_720nm_	FSEP-R_800nm_	FSEP-CI_rededge_	FSEP-CI_green_	FSEP-NDVI	FSEP-NDRE	FSEP-VARI	FSEP-MTCI	FSEP-EVI2
r	− 0.18^**^	− 0.23^**^	− 0.34^**^	0.75^**^	0.77^**^	0.68^**^	0.82^**^	0.80^**^	0.76^**^	0.70^**^	0.83^**^

** Correlation is significant at the 0.01 level (two-tailed)

### Rice LAI estimation using Fourier spectrum texture

**Fig. 7:**
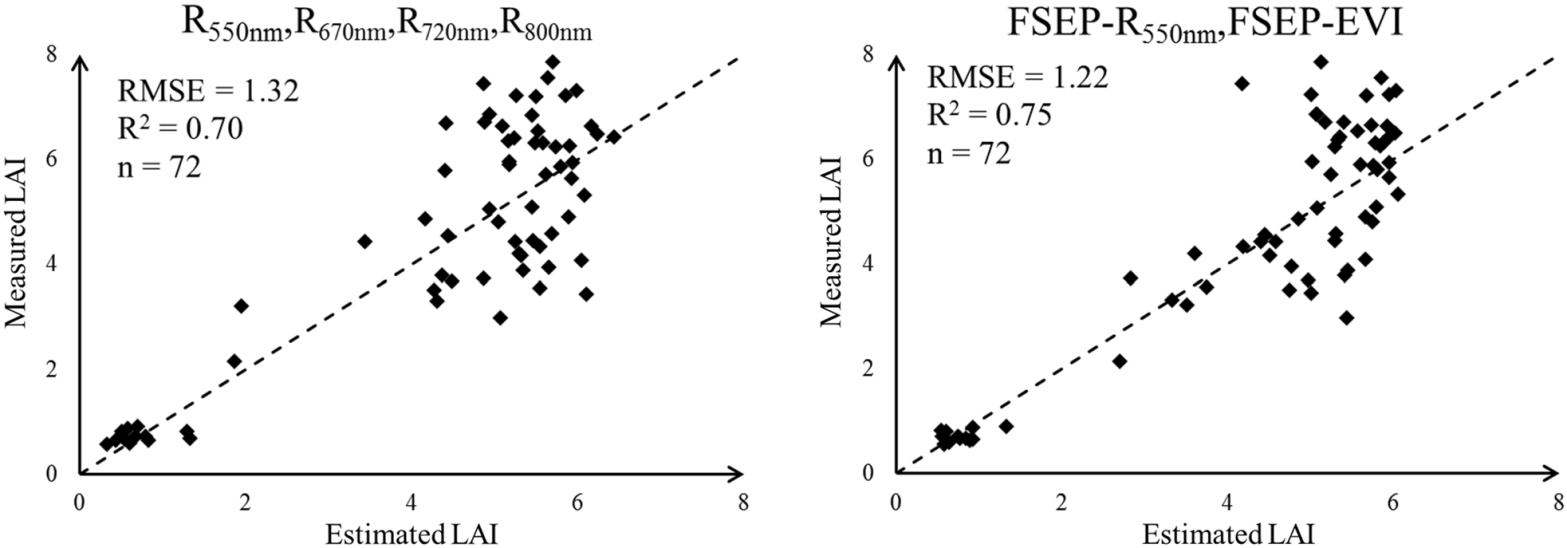
The relationship between estimated LAI and measured LAI

To determine whether FSEP can predict rice LAI more accurately, different predicted models were established by SVR with various parameters. The LAI samples were divided into two groups, one was used as the training set (n = 180) and the other one as the testing set (n = 72). The trained model was then applied in the testing set, the estimated LAI values were obtained and compared with the ground measured LAI values. RMSE and R^2^ of estimated LAI and measured LAI in the testing set were calculated to analyze the estimation precision of the trained model with different input parameters—Table 4. In general, the estimation precision of the FSEPs was higher than spectral parameters. Among all the tested input parameters, FSEP- R_550nm_, FSEP-EVI2 acquired highest estimation accuracy for LAI with the RMSE of 1.22. For reflectance parameters, increasing the count of same type parameter may not improve the estimation accuracy for LAI—the RMSE of R_550nm_, R_670nm_, R_720nm_, R_800nm_ was higher than that of R_550nm_, R_670nm_, R_800nm_. And the results showed that VIs did not perform better than reflectance bands in LAI estimation—the RMSE of R_550nm_, R_670nm_, R_720nm_, R_800nm_ was lower than that of NDRE, VARI, EVI2. A similar result was also found in the comparison of R_550nm_, R_670nm_, R_800nm_ and R_550nm_, EVI2. Compared with spectral parameters, FSEPs worked better for LAI estimation. Both VI-derived FSEPs and reflectance-derived FSEPs can be used as the input of SVR to develop LAI estimation model. The RMSE of FSEP-NDRE, FSEP-VARI, FSEP-EVI2 and FSEP- R_550nm_, FSEP-EVI2 was smaller than that of NDRE, VARI, EVI2 and R_550nm_, EVI2. These results suggested that the Fourier spectrum texture proposed in this paper was an effective indicator to estimate rice LAI—Fig. 7.

**Table 4:** The assessment of LAI estimation model established with different input parameters by SVR

Input parameter	Type	RMSE	R^2^
R_550nm_, R_670nm_, R_720nm_,R_800nm_	Spectral feature	1.32	0.70^**^
R_550nm_, R_670nm_, R_800nm_		1.30	0.71^**^
NDRE,VARI,EVI2		1.37	0.66^**^
R_550nm_,EVI2		1.29	0.72^**^
FSEP- NDRE,FSEP-VARI,FSEP-EVI2	Texture feature	1.23	0.76^**^
FSEP– R_550nm_,FSEP–EVI2		1.22	0.75^**^

** F-test statistical significance at 0.01 probability level

## Discussion

The primary purpose of this study was to improve the accuracy of rice LAI estimation based on the UAV image. A Fourier spectrum texture feature called Fourier spectral energy percentage (FSEP) was applied and proved to be a more effective indicator for rice LAI estimation.

The remote sensing spectral feature was regarded as a good indicator for estimating vegetation greenness-related parameters such as chlorophyll content, LAI and vegetation fraction [42]. Especially the VI, obtained by spectral transformation of several reflectance bands, was more useful in monitoring vegetation growth for its ability to enhance the vegetation feature [9]. In this study, the rice canopy reflectance and various VIs were firstly tested to correlate with rice LAI. Four reflectance bands were selected including R_550nm_, R_670nm_, R_720nm_ and R_800nm_, and the canopy reflectance in these bands was proved to be related with rice LAI [43, 44]. Besides, seven commonly used VIs based on these reflectance bands were also tested—Table 1. Previous studies showed that it was possible to estimate rice LAI directly from the reflectance of red and NIR region [45]. In this study, R_670nm_ and R_800nm_ also exhibited a stronger correlation with rice LAI than R_550nm_ and R_720nm_, and R_800nm_ produced the highest r value among all reflectance bands (r was 0.77). According to the characteristic of vegetation spectral curve, the near-infrared (NIR) spectrum of vegetation is related to the vegetation canopy structure [6]. LAI, calculated based on the leaf area of vegetation, was an important vegetation growth parameter about canopy structure [46] and thus R_800nm_ performed a stronger correlation with rice LAI. As for VI, all the tested VIs showed an acceptable correlation with LAI (r above 0.5), and NDVI and EVI2 were the most relevant among them (r was 0.79). Note that, NDVI and EVI were both calculated based on R_670nm_ and R_800nm_ which had a strong correlation with LAI. It implies that the relevance of reflectance bands may affect the corresponding VI’s correlation with LAI. The VI based on strongly correlated reflectance bands may perform a better correlation. It has been reported that NDVI saturated if plant canopy structure was complex [47]. Consequently, Fig. 4 showed that NDVI suffered from the obvious saturation effect in high LAI values. In this case, R_800nm_ and EVI2 were selected as the best spectral indices to estimate rice LAI among the tested reflectance bands and VIs. Although VI had a potential to enhance the vegetation feature compared with canopy reflectance, the correlation of LAI and EVI2 was not significantly better than that of LAI and R_800nm_. This result suggests that the simple spectral transformation of different reflectance bands may not meet the demand of more accurate LAI estimation.

In this study, the raw UAV images were converted into the reflectance images by radiance calibration and then we obtained various VI images based on band math. Moreover, the average of all pixel values in rice plot ROI was calculated as the plot level canopy reflectance and VI. Apparently, a simple average of related pixels may not contain the detailed texture of images which are sensitive to the shape, height, and size of the canopy [48], using only canopy reflectance and VI obtained by this way may limit their potential for LAI estimation. In order to describe the texture of the UAV image in rice field, Fourier transform was used to transform the image from the spatial domain into the frequency domain and the Fourier spectral energy percentage (FSEP) was gained as the texture feature.

Firstly, two binary images with regular stripes were simulated to analyze the relationship between FSEP and image texture. Two simulated images were of the same size and shape, and the stripes in two images were the same. There were both ten stripes in two simulated images, the position of center line of each stripe in two images was distributed uniformly and fixed at the same column. But the width of stripe was different in two images. In addition, the pixel values which belonged to stripe were set to 1 and the other pixel values were set to 0—Fig. 5a, b. These two images were designed to simulate the rice growth in field. The rice plant was transplanted line by line, and the position of rice plant was fixed after transplant. So, the rice plant line was like the stripe and the stripe width become wider and wider with rice growth. Moreover, LAI was the major contributor to make stripe wider. After Fourier transform, the Fourier energy spectrum image of two simulated images was calculated by Eq. (6)—Fig. 5e, f. The Fourier energy spectrum image was stretched to enhance the chiaroscuro and the bright spots in energy image implied the high energy value. Results showed that the high value distribution of the energy spectrum was related to the width of stripe. The wider the stripe, the more concentrated on center of the high energy value was. To verify the practicability of simulation results, the method above was applied in real UAV images. Focused on the same rice plot, we obtained two EVI images collected on two different days. These two EVI2 images were clipped into the same size and shape, and one was based on the UAV image taken on February 4th and the other one was on March 9th—Fig. 5c, d. Obviously, the rice on March 9th had higher LAI value and thus the EVI2 image collected on March 9th may have wider stripes. Fortunately, the same phenomenon was also found in real UAV images. The high energy value of the Fourier energy spectrum produced by the EVI2 image with wider stripes was more concentrated on center point—Fig. 5g, h.

The simulated image and the real UAV image both showed that the high value distribution of energy spectrum was related to rice LAI. As for the Fourier energy spectrum image, the total energy of whole image was constant [39]. If the high energy value was concentrated on center region, the sum energy of center region would take up a large proportion in total energy. Therefore, the energy spectrum was separated by some rectangle rings—Fig. 3d, and the percentage of center ring energy above the total energy of all rings was calculated as FSEP value to represent the texture feature. The higher the FSEP value, the more concentrated on center of the high energy value was. In this way, the relationship between FSEP and LAI was established.

To investigate the relationship between FSEP and rice LAI, we calculated a series of FSEP based on different reflectance and VI images. And the correlation analysis and regression analysis were utilized. The results of correlation analysis showed that most reflectance-derived FSEPs had a relatively weak correlation with LAI except FSEP-R_800nm_. The goodness of fit between LAI and FSEP-R_800nm_ can reach 0.69—Fig. 6. That may be because that the contrast between vegetation and background is more obvious in NIR reflectance band [49]. If the contrast between vegetation and background was more obvious, the texture of image would be clearer and thus FSEP would be more sensitive to rice LAI. VI had the better ability to enhance the contrast between vegetation and background [50], and thus VI-derived FSEPs exhibited a significantly stronger correlation with LAI than reflectance-derived FSEPs– Table 3. Although the correlation of LAI and EVI2 was not significantly better than that of LAI and R_800nm_, the correlation of LAI and FSEP-EVI2 was quite stronger than that of LAI and FSEP-R_800nm_. Compared with VI, the VI-derived FSEP exhibited a stronger correlation with LAI. However, the FSEP texture cannot prevent NDVI suffering from saturation—Fig. 6.

To determine whether FSEP can predict rice LAI more accurately, different predicted models were established by SVR. FSEPs and some other spectral parameters were employed as the inputs of SVR respectively—Table 4. The tested input parameters contained three types of parameter, including reflectance type, VI type and FSEP type. Reflectance type and VI type belonged to spectral features, and FSEP type belonged to texture features. For reflectance data, band selection was essential to improve the training efficiency of machine learning technology [51]. In this study, the estimation accuracy of R_550nm_, R_670nm_, R_800nm_ was higher than that of R_550nm_, R_670nm_, R_720nm_, R_800nm_. It suggests that choosing the appropriate reflectance bands was more important than increasing the count of input reflectance bands. One VI could contain the information of two or three reflectance bands. But a simple spectral transformation cannot help to improve the accuracy of LAI estimation in SVR training model—the RMSE of R_550nm_, R_670nm_, R_720nm_, R_800nm_ was 1.32 and the RMSE of NDRE, VARI, EVI2 was 1.37. The reason may be that the SVR itself has an ability to find the suitable combination of different reflectance bands. These results imply that VI used as the input of SVR may not establish a better estimation model than canopy reflectance. Thereby, the texture feature was considered and the result proved that FSEPs could estimate rice LAI more accurately. Compared with NDRE, VARI, EVI2 and R_550nm_, EVI2, FSEP-NDRE, FSEP-VARI, FSEP-EVI2 and FSEP- R_550nm_, FSEP-EVI2 performed better in rice LAI estimation. It means that the FSEP extracted from VI images and reflectance images can be as the input of SVR to improve LAI estimation in rice—Fig. 7.

## Conclusions

In this study, we developed a Fourier spectral energy percentage (FSEP) method to improve the accuracy of rice LAI estimation based on the UAV image. The Fourier energy spectrum of simulated images and real UAV images implied that there was a strong relationship between FSEP and rice LAI. And the result of correlation analysis showed that the VI-derived FSEP had stronger correlation with rice LAI than VI. When used as the input parameter of SVR, FSEP could also obtain more accurate estimation model of rice LAI than VI. Therefore, the texture feature of high-resolution remotely sensed images can be a more effective indicator for estimating vegetation growth parameters.

Although the texture feature proposed in this study were tested in rice, this work may offer a theoretical framework for vegetation growth parameters estimation in crops which have obvious detailed texture in high resolution images. In the future study, we will try to apply this approach in other crops.

## Data Availability

Not applicable.
